# P-1175. Healthcare-Associated Respiratory Viral Infection Outbreak in a Neonatal Intensive Care Unit

**DOI:** 10.1093/ofid/ofae631.1361

**Published:** 2025-01-29

**Authors:** Elizabeth Marrero, Michele Honeycutt, Jennifer Henthorne, Kristin Pool, Ana Del Valle Penella

**Affiliations:** Arkansas Children's Hospital, Little Rock, Arkansas; Arkansas Children's Hospital, Little Rock, Arkansas; Arkansas Children's Hospital, Little Rock, Arkansas; Arkansas Children’s Hospital, Little Rock, Arkansas; University of Arkansas for Medical Sciences / Arkansas Children's Hospital, Little Rock, Arkansas

## Abstract

**Background:**

Healthcare-associated respiratory viral infections (HARVIs) have the potential to cause considerable harm including increased morbidity and mortality in patients in neonatal intensive care units (NICUs). We report an HAVRI outbreak in an open pod 104 bed Level IV NICU at a standalone children’s hospital in the south-central region of the United States and the control measures implemented to contain the outbreak.

**Figure:**
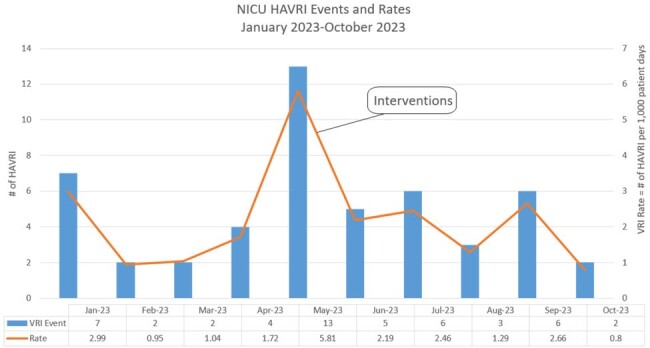

**Methods:**

An outbreak was identified through a 255% increase in HARVI rates over four months (figure 1). An interdisciplinary team was formed to address the outbreak.

Interventions included: 1) universal masking, 2) isolation of positive patients and pod-mates with a total of three pods being closed for a total of two weeks from the date of last exposure, 3) visitor restrictions with symptom screening at unit entrance, 4) enhanced environmental measures such as equipment cohorting, baseline deep clean, and increased cleaning frequency, 5) infection prevention practices such as hand hygiene, bare below the elbows, and isolation practices were reinforced with the staff.

**Results:**

Post-interventions, the HARVI rate dropped by 70% over a five month period. During the outbreak, several patients experienced longer hospital stays, three patients required escalation of care, and no deaths occurred (Figure 1).

**Conclusion:**

Rapid identification and response are crucial in managing HARVIs in NICUs. The collaborative efforts between NICU leadership, infection prevention, and hospital administration played a key role in promptly controlling the outbreak.

**Disclosures:**

**All Authors**: No reported disclosures

